# Projecting health-ageing trajectories in Europe using a dynamic microsimulation model

**DOI:** 10.1038/s41598-021-81092-z

**Published:** 2021-01-19

**Authors:** Guillaume Marois, Arda Aktas

**Affiliations:** 1 Asian Demographic Research Institute, School of Sociology and Political Sciences, Shanghai University, 99 Shangda Rd., Shanghai, 200444 China; 2 Wittgenstein Centre for Demography and Global Human Capital (IIASA, OeAW, University of Vienna), International Institute for Applied Systems Analysis, Schlossplatz 1, 2361 Laxenburg, Austria

**Keywords:** Quality of life, Epidemiology

## Abstract

The extent of the challenges and opportunities that population ageing presents depends heavily on the population’s health. Hence, for the development of appropriate strategies that enable countries to adopt the emerging demographic and epidemiological realities, information on future health trajectories of elderly population is a natural requirement. This study presents an innovative methodological framework for projecting the health of individuals using a dynamic microsimulation model that considers interactions between sociodemographic characteristics, health, mortality, bio-medical and behavioral risk factors. The model developed, called ATHLOS-Mic, is used to project the health of cohorts born before 1960 for the period 2015–2060 for selected European Countries using SHARE data to illustrate the possible effects of some selected risk factors and education on future health trajectories. Results show that, driven by a better educational attainment, each generation will be healthier than the previous one at same age. Also, we see that, on average, an individual of our base population will live about 18 more years since the start of the projection period, but only 5 years in good health. Finally, we find that a scenario that removes the effect of having a low level of education on individual health has the largest impact on the projected average health, the average number of years lived per person, and the average number of years lived in good health.

## Introduction

Over the last century, improvements in living conditions and medical progress have significantly increased the average life expectancy, as well as the chance of survival to older ages^1–3^. Today, globally, a person aged 65 years expects to live, on average, additional 17 years, and by 2050 this figure is projected to be 19 years^4^. However, these gains in the average length of life, along with lower fertility rates, have led to an unprecedented and continuing increase, both in the number and the proportion, of older people in the population^4^. According to the World Population Prospects 2019 of the United Nations^4^, the global population of people aged 65 years and over is expected to more than double, from 703 million to 1.5 billion by 2050, while the share of old people in the global population is projected to rise from 9 to 16%.

As most countries are going through a transition towards an older population structure at different rates^4^, there is growing recognition among scientists and policy makers that the extent of the future opportunities and challenges that arise from the aging population will be heavily dependent on the health status of older people^5–7^. If the gains in longevity are accompanied by low levels of functional and cognitive capacity, as well as increases in diseases and disease-related disabilities, they will not only affect the wellbeing of individuals negatively, but also put pressure on the functionality and fiscal sustainability of the systems designed to support older people^7^ such as pension, social insurance, public health, and long-term care systems. Conversely, if these additional years are spent in good health, the implications can be much more positive^7^, both at the individual and the societal level. Multiple studies^8–13^ suggest that people who can maintain good health and functional ability at older ages may stay active in the labor force for a longer period of time, and hence, keep contributing to the economy by paying taxes and social security contributions, saving, consuming and even increasing the average labor productivity. Moreover, literature has emphasized that well-being and ageing are closely linked to mental and physical health^14,15^. Given the paramount importance of the successful promotion of healthy ageing, the identification of trajectories of health state of older people is central to estimate the needs of the aging population accurately and to formulate effective and versatile policies which aim to ensure the well-being of elderly people, as well as the viability of the social, economic and political systems in the future^16^.

Identifying future health trajectories is challenging for several reasons. First, health is a complex and multidimensional phenomenon characterized differently under multiple definitions^17^. In the absence of a standard approach to measuring health, researchers have used various health indicators depending on the purpose of the study as well as the availability of data. Some examples are self-assessment of measures of health-state (i.e., self-rated health), measures of the ability to function independently (e.g. ADLs and IADLs), morbidity indicators (e.g. whether an individual is or has suffered a particular disease) and mortality rates^18,19^. However, none of these indicators can comprehensively and objectively evaluate a person’s true health status, as some of them suffer from certain degrees of reporting error^20,21^ or epistemic problems associated with the either objective or subjective discretization of health in different categories^22^. Moreover, the amount of variations in the measurement of health across different studies creates significant difficulties in obtaining a comparable set of health information that can be adapted for international or inter-group comparisons^23^.

Another challenge arises from the fact that health is a product of an array of social, behavioral, environmental, cultural, and biological factors, as well as their complex and dynamic relations at different levels^24^. Therefore, age-related changes in health, despite the well-established association between advanced age and the increased risk of bad health (as measured in terms of prevalence of non-communicable diseases^25^, cognitive impairments^26,27^ and physical frailty)^28–30^, are not uniform across individuals of the same age, but, on the contrary, vary greatly depending on individual-level characteristics and health-related risk factors. Because of such complexities, the identification of future health trajectories requires a micro approach which allows for the simulation of individual health patterns through time, taking individual attributes (e.g., age, sex, education level, heath habits, health-related risk factors), history (e.g., previous years’ heath state and health habits), as well as the dynamic interaction among these factors into consideration. However, conventional population projection methods, such as the cohort-component model, are limited in their ability to incorporate these important sources of population heterogeneity beyond age, sex, and few additional dimensions, such as region or broad education categories into the projections^31^. Therefore, cohort-based models are not useful for the study and projection of population health-ageing trajectories, taking individual-level heterogeneity into the consideration.

On the other hand, dynamic microsimulation models can be an appropriate modelling choice for simulating the evolution of complex dynamics resulting from many simultaneous processes^32,33^, as it is the case for health status. This is because, different from traditional cohort based model, dynamic microsimulation models describe the events and outcomes at the individual-level, keeping the records of individual histories over the life time and allowing the inclusion of a large number of dimensions of interest without imposing limits to variable types^31^. Therefore, these models can project the individual-level health trajectories more accurately than the other modelling options do^34^. Moreover, this framework can be used to simulate the impacts of prospective policies and programs on the individual health trajectories^32,35^. Overall, the dynamic microsimulation modelling framework is an advanced but flexible tool that shows adequacy for the assessment of the population aging trajectories on the basis of health, across population subgroups within and across countries.

Based on these underpinnings, the objective of this paper is to present a demographic modelling framework to project the trajectories of health status of individuals with a set of health-related risk factors using a dynamic microsimulation model. The model presented, called ATHLOS-Mic, aims to implement dynamically the effects of health-related risk factors and socioeconomic characteristics and their interactions on health and mortality trajectories of the elderly population in Europe from 2015 to 2060. In the model, the health at older ages is measured using a novel health metric, developed and validated as a part of the ATHLOS project in Caballero et al.^36^ and De la Fuente et al.^37^, which consolidates individual health status from a vector of functioning in different domains that have been selected according to the functional ability framework provided by the WHO^38^. In this sense, this measure has the advantage of displaying multiple aspects of health in a single continuous variable, and, unlike other health measures used in the simulations of health trajectories, it doesn’t suffer from some of the aforementioned data-related issues (e.g. epistemic problems, self-reporting error biases), neither does focus on single aspects of health (such as morbidity indicators).

This study contributes the literature by providing a population projection framework for forecasting population health trajectories taking into consideration individual-level heterogeneity as well as dynamic interactions between bio-medical and behavioral risk factors, health, and mortality. Using a dynamic microsimulation model and a novel health metric defining health as a single-continuous variable on the basis of functioning in different domains, our framework successfully overcomes the aforementioned difficulties arising from the complex structure of health and the methodological limitations of traditional population projection models. ATHLOS-Mic enriches the overall quality of population projections by allowing the incorporation of a health dimension into the population projections and by considering more sources of heterogeneity within groups. Therefore, the methodological framework presented in this study provides a very useful tool to gain insights on the determinants and dynamics of health-age trajectories, as well as for policy makers due to its ability to measure the effect of changes along several dimensions and allowing for a wide array of “what if” scenarios.

Although there are many microsimulation models that include health^39,40^, they generally focus on a specific set of diseases (e.g. heart diseases, diabetes, cancer and others in POHEM^41^) or use health measures that exhibit the deficiencies that have already been mentioned, such as self-reported health (e.g. DYNASIM3^42^), categorical health variables (e.g. good/bad health in SAGE^43^) or proxy variables (e.g. disability in DYNAMOD^44^). Moreover, these models are used for different purposes altogether, namely either epidemiological analysis or to assess their impact on a specific outcomes, such as mortality or public finances, disregarding the long term forecasting dimension from population projections^45–47^. To our knowledge, the model described in this study is the first population projection model that includes the forecasting of health using a functionality-based continuous health metric, as well as related risk factors, in addition to other standard demographic components.

The remainder of the paper is organized as follows. The next section provides a short summary about the microsimulation approach to modelling, and then describes the data sources, samples, key measures and design of the ATHLOS-Mic microsimulation model. “Simulation results” section presents some outcomes and a sensitivity analysis from different scenarios and “Discussion and conclusion” section provides discussion and concludes.

## Building ATHLOS-Mic microsimulation model

### Microsimulation models and their use in population projections

Microsimulation is an approach which allows for the simulation of the behavior of micro-units to describe the behavior of a system which cannot be easily understood at an aggregate level. Microsimulation models can be broadly divided into two categories: dynamic and static models, depending on whether micro-units are allowed to evolve over time. In a dynamic microsimulation framework, the baseline population consists of individuals whose characteristics represent the composition of a given population across a set of chosen dimensions. Each micro unit in the sample evolves individually over the modelled period. At each stage, individuals are exposed to the risk of a set of events which are relevant to their current state and characteristics. The transition between different states through the model is determined stochastically with a random experiment (i.e., Monte Carlo method). The inclusion of stochastic elements leads to different simulation outcomes for each individual, therefore allowing for an adequate representation of uncertainty and risk^48^. Moreover, microsimulation allows for flexible aggregation, i.e., simulation results, which have been generated independently for each individual, can be flexibly summed up based on any policy of interest at the reporting stage^49^. Microsimulation models have been considered as a powerful tool if the interested dynamic or behavior can be better understood at the micro level than at the macro level, or when the population heterogeneity or individual life history are important for ensuring the internal consistency of the model and simulation outputs^35,49^.

The concepts of microsimulation have been first introduced in Orcutt^50^ and Orcutt et al.^51^. Since then, different types of microsimulation models have been developed and used to address different types of research questions in various fields. For example, they have been used to evaluate the future performance of long term programs (e.g., pensions^52^, long-term care^53^, education); to simulate the potential impacts of prospective public policies or policy changes; to project life-time behaviors (e.g., saving, labor force participation) or complex dynamics (e.g., ageing) for policy analysis (an exhaustive overview of microsimulation applications in social sciences and other areas can be found elsewhere^32,35^). The recent developments in computing technology, as well as the rise in the number of micro-data sources needed to calibrate the parameters of the microsimulation, have made it easier to develop more complex models and also increased the level of interest for such models^54^.

The use of microsimulations for population projections is relatively new, even though the advantages of such models compared to the traditional macro-level population projection models have been discussed for decades^31^. Microsimulation methods have been used by population scientists to model demographic processes, to make detailed and realistic population projections encompassing various population dimensions, and to gain insights on life course transitions^55^. Some examples of the use of microsimulation models in demography are the following: projection of the future effects of the ethnocultural diversity^56,57^; simulation of the changes in human-capital accumulation, immigration volumes, speed of integration, and labor force participation^58^; forecasting the potential impact of sociocultural inequalities in education on the future human capital accumulation level^59^; simulation of the impacts of the decline of literacy skills on the future working-age population; and projection of populations in small areas^60^. See Lomax and Smith^61^ for a good overview of microsimulation applications in demography.

Despite their requirement for high computing power and technical capability, microsimulation models are flexible and adaptable tools that allow for doing many types of experiments and scenario-based studies, therefore they are useful for researchers and policy makers.

### Data

A consistent and rigorous modeling of health for projection purposes requires parameters that can be used to model the relationship between individual’s health and its sociodemographic, bio-medical and behavioral determinants. In consequence, modeling relies on the availability and quality of individual-level data representative of the target population. The ATHLOS-Mic model utilizes data from the Survey of Health, Ageing and Retirement in Europe (SHARE), an individual-level longitudinal dataset including detailed information on a wide variety of aspects, among them health status, wellbeing, and socio-economic characteristics of people aged 50 or older in the selected European countries. The large set of health and socio-demographic information that SHARE provides makes it especially suitable for our purposes (see Section S.2.1 for the discussion on the limitations of SHARE). In particular, we use a version of SHARE available as part of the harmonized data set (HD) that has been compiled in the context of the ATHLOS project (henceforth called SHARE-HD). The HD standardizes a set of health-related variables provided by several international and national surveys comprising a health metric corresponding to the definition that will be stated in the next section. In this way, the variables found in the HD are comparable in their definition and share their reference formats among the different surveys (see Sanchez-Niubo et al.^62^ for details of ATHLOS-HD).

Our particular sample is derived from four out of five waves of the SHARE-HD, covering the period from 2004 to 2014. It excludes the third wave (i.e., the year of 2008) since it doesn’t include some of the variables of interest. Among the 27 countries available in SHARE-HD, only 14 countries are selected for the final sample, as they have at least two waves of data collection in two consecutive years, which is the minimum requirement for the estimation of transition rates. The final sample hence consists of individuals aged 50 older who have observed values for all variables and covariates of interest in at least two consecutive waves. The sample sizes by country and wave are given in Table 1. The proportion of missing data is lower than 3% in all variables that have been used, and therefore no imputation was required.

**Table 1:** Sample size by country and wave (in parenthesis, the retention rate from previous wave, including deceases).

Country	ISO Code	Wave 1 (2004)	Wave 2 (2007)	Wave 4 (2011)	Wave 5 (2013)
Austria	AT	1594	1228 (72%)	5332 (56%)	4425 (76%)
Belgium	BE	3827	3205 (74%)	5388 (69%)	5765 (75%)
Czech Republic	CZ		2830	6196 (49%)	5926 (69%)
Germany	DE	3008	2614 (53%)	1623 (54%)	5719 (65%)
Denmark	DK	1707	2666 (76%)	2393 (68%)	4268 (85%)
Estonia	EE			6828	6064 (85%)
Spain	ES	2396	2315 (61%)	3690 (65%)	6690 (80%)
France	FR	3193	3021 (64%)	5954 (65%)	4588 (68%)
Greece	GR	2898	3292 (80%)		
Italy	IT	2559	3039 (71%)	3673 (68%)	4853 (73%)
Netherland	NL	2979	2710 (61%)	2822 (62%)	4213 (80%)
Poland	PL		2467	1880 (67%)	
Sweden	SE	3053	2802 (68%)	2122 (60%)	4713 (72%)
Slovenia	SI			2756	3000 (73%)
Total		27,214	32,189 (68%)	50,657 (63%)	60,224 (71%)

Source: Authors' calculation using data from SHARE-HD.

### Design and components

The ATHLOS-Mic is built over another microsimulation projection model, namely, CEPAM-Mic, which already has core modules to perform a multidimensional population projection by various population characteristics. The Centre for Expertise on Population and Migration (CEPAM) aims to investigate the impacts of immigration on the future population in European Union. Full details of the architecture of CEPAM-Mic are available elsewhere^54,56,59,63,64^. In brief, CEPAM-MIC is a dynamic, continuous time, event-based, and spatial microsimulation projection model programed in the Modgen language, a microsimulation programming language developed by Statistics Canada, integrated into the Microsoft Visual Studio C +  + environment. CEPAM-MIC provides the basic functions and elements for microsimulation (e.g., actors, states, events)^54^. In addition, yearly probabilities that are either directly implemented or calculated from regression parameters implemented as inputs through a user-friendly interface, are posteriorly adjusted for a continuous-time modeling using a time function. The transitions between states are determined stochastically using a random experiment (i.e., Monte Carlo method). When an individual dies or emigrates, its simulation stops.

Building ATHLOS-Mic over CEPAM-Mic instead of directly simulating the SHARE-HD dataset has two main advantages. First, it allows us to rely on an already validated model for the demographic components of the simulation (in particular, the assumptions on future life expectancy). Second, it allows the simulation of a much larger sample (i.e., there are 8,148,874 individuals in the base sample of CEPAM-Mic, of which 2,367,443 meet the age and country criteria for ATHLOS-Mic.) that is representative of the population by age, sex, education and country of residence. Simulating such a large sample reduces drastically the Monte Carlo error and eliminates the need of performing many runs of simulation to get consistent and robust results.

**Figure 1:**
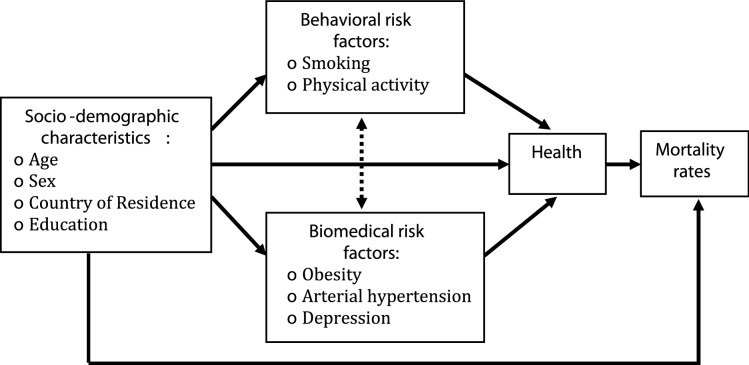
Schematic representation of the interaction among socio-demographic characteristics, risk factors, health and mortality in the ATHLOS-Mic’s health module.

ATHLOS-Mic is developed by implementing a health module into CEPAM-Mic. The framework of the ATHLOS-Mic’s health module is schematized in Fig. 1. Using sociodemographic characteristics and a set of risk factors, the newly developed health module uses parameters from statistical models to project the health of cohorts born before 1961 for a selection of European countries up to the 2060 horizon. SHARE-HD only surveyed the population aged 50 and over. Since the simulation starts in 2011, cohorts born after 1961 cannot be included as we don’t have data on their health characteristics. Therefore, there are no new entries into the simulation sample from the aging of younger cohorts. The level of health is also used to modulate the probability of survival. The health module is thus dynamically implemented into the model, allowing it to impact the projection outcomes in other dimensions.

In the model, individual health is measured using a novel composite index of individual health (henceforth HM) which has been developed and validated in Caballero et al.^36^ as a part of the ATHLOS project. The construction of this metric treats a person’s true health status as a latent variable to be inferred from detailed survey data on self-reported health indicators as well as physical and cognitive tests. More specifically, using Bayesian Item Response Theory, the distribution of a health score is estimated in a way that reflects the distribution of the observed health status (represented by the health-related characteristics) of a particular sample. In this sense, if two individuals have the same health score, they will have the same probability of exhibiting a particular health characteristic, but they may not necessarily present the health characteristic at the same time (see S1 in the Supplementary Materials for more details on the health metric).

Under this approach, health is characterized as a vector of functioning in different domains, ranging from simple to complex (e.g., vision, walking, kneeling, Activities of Daily Living, Instrumental Activities of Daily Living). The approach conceptualizes the notion of health within the functional ability framework suggested by the World Health Organization (WHO)^38^, therefore focusing on function (i.e., capabilities which enables people to meet their basic needs; learn, grow, and make decisions; be mobile; build and maintain social relationships; and contribute to society) rather than on specific diseases or comorbidity. The novelty of this health metric lies chiefly in the ability to simultaneously capture many aspects of individual health in a single measure. Additionally, unlike most other measures commonly used in the literature, this health metric is obtained as a continuous variable (ranging from 0 to 100), and therefore does not suffer from the epistemic problems associated with either the objective or subjective discretization of health in different categories (e.g. self-rated health or measures of the ability to function in daily activities, such as Activities of Daily Living (ADLs) and Instrumental Activities of Daily Living (IADLs). See Benitez-Silva and Ni (2008) for a detailed analysis of these measures).

Table 2 shows the variables included in ATHLOS-Mic. Sociodemographic characteristics used include age, sex, country of residence and level of educational attainment. The risk factors included into the module are chosen among those which are found to be associated with health in the related literature. Furthermore, the set was reduced to 5 factors due to data limitations in SHARE-HD, and posteriorly classified in two categories: behavioral factors which includes smoking status and physical activity, and bio-medical factors including obesity, arterial hypertension and depression status (see section S.2.2. for details).

**Table 2:** Variables in the ATHLOS-Mic.

Variable	Event Type	Definition, Categories
**Socio-demographic characteristics**		
Age	Deterministic	In years, continuous, + 50
Sex	Invariable	Male, female
Country	Stochastic	Country of residence, 14 categories
Education	Invariable	Divided into 3 categories based on ISCED classification: Low: lower secondary or less (ISCED 1, 2); Medium: upper secondary completed (ISCED 3); High: post-secondary and more (ISCED 4 +)
**Behavioral risk factors**		
Smoking	Stochastic	1 if the individual is current smoker; 0 otherwise
Physical activity	Stochastic	1 if the individual did vigorous physical activity in the last two weeks; 0 otherwise
**Biomedical risk factors**		
Obesity Status	Stochastic	Based on Body-Mass-Index (BMI) derived from self-reported weight and height: 1 if BMI ≥ 30 kg/m^2^; 0 otherwise
Arterial hypertension (AH)	Stochastic	1 if the individual suffers from AH the at the survey time; 0 otherwise
Depression Status	Stochastic	1 if the individual suffers from the depression at the survey time; 0 otherwise
**Health outcomes**		
Health	Deterministic	Continuous, take a value in [0–100]
Survival	Stochastic	1 if alive; 0 if dead

### Methodology

The ATHLOS-Mic is built over CEPAM-Mic in 5 steps:

#### Step 1: Imputation of initial health and risk factors to the base population

The first step is to set initial values for the health metric and risk factors. The base population of CEPAM-Mic corresponds to the year 2011, of which 2,367,443 individuals meet the age (cohort < 1961) and country criteria for ATHLOS-Mic. The imputation of risk factors to the base population of CEPAM-Mic, already including a large set of sociodemographic characteristics (e.g. age, sex, education, country of residence), is performed using microdata of the 4th wave of SHARE-HD, also corresponding to the year 2011. For the imputation, polytomous logistic regressions from the MICE package in R^65^ are used. This method performs multiple simultaneous imputations based on the observed values of selected covariates, thus allowing the correlation among imputed variables to be taken into consideration. Posteriorly, parameters from a linear regression modeling the logit of the health metric by sociodemographic characteristics and risk factors are used to impute an initial value of the health metric.

The Pearson correlation coefficients between the imputed variables in the base population and the data used as source for the prevalence of each risk factors and the distribution of the health metric, grouped by age, sex, country of residence and educational attainment, are all above 0.95 (Tables S1 and table S2), with no notable differences for older age groups. Pertaining the proportion of each imputed variable by other imputed variables, the correlation coefficients between the two datasets are also all above 0.9. In addition, we also performed Mincer-Zarnowitz regressions^66^ for each of the imputed variables to test for systematic biases. Parameters are all very close to 1 (table S3), indicating there are no biases. Therefore, we have that, for every set of covariates, the average imputed value in the base population corresponds approximatively to the value of the SHARE-HD population with the same set of covariates.

#### Step 2: Modeling the changes in sociodemographic characteristics

ATHLOS-Mic uses the core modules of CEPAM-Mic to perform a multistate and multiregional population projection by age, sex, education, and country of residence. Given that our target population would be over age 55 at the initial year of the projections, only the mortality module of CEPAM-Mic affects the population composition of the health module. The main assumption here is that the upward trend in life expectancy continues, but at different rate, by country, gender, and level of education.

#### Step 3: Modelling the changes in behavioral and bio-medical risk factors

The initial values set to individuals of the base population for behavioral and bio-medical risk factors are allowed to evolve over the projected period. To model their evolution, we calculated transition rates using an autoregressive distributed lag (ARDL) model, which accounts for multiple lags of the independent variables as well as multiple lags of the dependent variable. The equation is the following:

1 $$ logit\left( {F_{t} } \right) = \beta_{0} + \beta_{1} \left( {a - 1} \right) + \beta_{2} F_{t - a} + \beta_{i} X_{i, t - a} $$

where ${F}_{t}$ is the value of the risk factor at time *t*, *a* is the number of years between two observations and ${X}_{i, t-a}$ is a set of $i$ covariates $X$ (i.e., risk factors and sociodemographic characteristics) at time $t-a.$ The term ${\beta }_{1}(a-1)$ is included to control for the varying length between years of interview, assuming a linear distribution over time. When the duration is 1 year, ${\beta }_{0}+{\beta }_{2}$ can be interpreted as being the net probability of still having a factor $F$ for the individuals who have already had it 1 year ago. In the estimation we used all SHARE-HD wave pairs stacked up. As more than one observation for an individual may be included in the database (i.e., the change between wave 1 and 2 and the change between waves 4 and 5), the models are estimated using Generalized Estimating Equations (GEEs)^67^, which take into account the correlation between outcomes due to the availability of multiple observations per individual in the sample. The estimated parameters are presented in Table S4. The percent of concordance and the C-statistic are show in Table S5. For the 5 models, both indicators have very high values, close to or above 0.8, indicating that models have a strong predictive power.

The results show a strong interrelation between risk factors. For instance, the odd of doing physical activity at time $t$ is strongly decreased if the individual at time $t-a$ was a smoker (− 0.261), was obese (− 0.333), or was depressed (− 0.399). Similarly, it is more likely that individuals would be depressed at time $t$ if they were smokers (0.197) or obese (0.125) at time $t-a$; but it is less likely if they used to do physical activity at time $t-a$ (− 0.234). The only risk factor that seems purely autoregressive is smoking, as all the corresponding parameters for other risk factors are not significant.

The level of education has a considerable influence on other risk factors, regardless of it being almost fixed for the elderly population. In fact, having a low level of education substantially increases the risk of obesity (0.692), depression (0.493) and arterial hypertension (0.313), while reducing the chance of doing physical activity (− 0.244). On the other hand, the coefficients for education are not significant in the smoking model, implying that the likelihood of quitting smoking for elderly individuals is the same regardless of their level of education (although this doesn’t mean that the probability to start smoking at a younger age is not influenced by the education level).

Last but not least, age is highly associated with the risk factors (see Figure S1). In fact, the chance of keep smoking, being obese and doing physical activity decreases with age while the chance of suffering from depression increases even faster with age, particularly after age 75. On the other hand, the risk to start developing arterial hypertension increases by age, reaches its peak by age 75, and then declines.

The estimated coefficients are used to derive the net probability of yearly change in the risk factors for a standardized profile of an individual living in one of the EU countries in our sample. The net transition rates from time (*t*−1 to *t*) for all risk factors and individual characteristics are presented in Table S6.

Using the initial values of the risk factors imputed to the base population ${F}_{t}^{^{\prime}}$ as described in step 2 in conjunction with the estimated parameters in this step, Monte Carlo simulations are performed to predict stochastically the value of the risk factors at time $t+1$ throughout the projection period (see Eq. S1 for the formula).

#### Step 4: Modeling the change in health

The health module of the microsimulation model generate changes in the health of the individuals through the simulation period as it evolves over the life course in line with the evolution of the characteristics and the risk factors. Using the aforementioned health metric (HM), the change in health is modelled in two steps. First, given that the health metric is a continuous variable ranging from 0 to 100, we use a linear regression model on the difference between the logit of the values of the health metric over a year, as expressed in Eq. (2):

2 $$ \begin{aligned} & \frac{{logit\left( {\frac{{HM_{t} }}{100}} \right) - logit\left( {\frac{{HM_{t - a} }}{100}} \right)}}{a} \\ & \quad = \beta_{0} + \beta_{1} \frac{{HM_{t - a} }}{100} + \beta_{2} \left( {\frac{{HM_{t - a} }}{100}} \right)^{2} + \beta_{3} \left( {\frac{{HM_{t - a} }}{100}} \right)^{3} + \beta_{i} X_{i, t - a} \\ \end{aligned} $$

where ${\beta }_{i}$ is a set parameters capturing the effect of covariates $X$ at time $t-a$ (i.e., sociodemographic characteristics and risk factors). ${\beta }_{1}$ + ${\beta }_{2}$ + ${\beta }_{3}$ allows for different rates of change in the health metric based on the initial health status. We used the logit of the health metric in order to avoid negative outcomes or outcomes exceeding 100. Since what we model is the change in the health metric, the initial value of the health metric is also implemented as a predictor, as the decline in health is likely to be different for people who are initially in bad or good health. The model is estimated using generalized estimating equations (GEE) and its results are presented in Table S7 (also see Figure S2 for comparison of the predicted and observed changes in the logit of the HM over one year).

As expected, all risk factors increase the pace of the decline in the health. After controlling for other risk factors, education still has a strong impact, indicating it accounts as a proxy for many other sources of social inequalities and heterogeneity that are not included in the model. The health was also found to decline faster for people in good health than for those in medium or bad health.

Hence, starting from the initial values of the health metric (HM’), we estimate the predicted value of the HM at time t + 1 using the estimated parameters from Eq. (2), as expressed by Eq. (3):

3 $$ HM_{t + 1}^{^{\prime}} = 100*\frac{{{\text{exp}}\left( {\frac{{logit(HM_{t}^{^{\prime}} )}}{100}} \right) + \beta_{0} + \beta_{1} \frac{{HM_{t}^{^{\prime}} }}{100} + \beta_{2} \left( {\frac{{HM_{t}^{^{\prime}} }}{100}} \right)^{2} + \beta_{3} \left( {\frac{{HM_{t}^{^{\prime}} }}{100}} \right)^{3} + \beta_{i} X_{i, t - a} }}{{1 + {\text{exp}}\left( {\frac{{logit(HM_{t}^{^{\prime}} )}}{100}} \right) + \beta_{0} + \beta_{1} \frac{{HM_{t}^{^{\prime}} }}{100} + \beta_{2} \left( {\frac{{HM_{t}^{^{\prime}} }}{100}} \right)^{2} + \beta_{3} \left( {\frac{{HM_{t}^{^{\prime}} }}{100}} \right)^{3} + \beta_{i} X_{i, t - a} }} $$

In this way, the future value of the health metric is calculated deterministically, although the variables used in its prediction were computed stochastically.

#### Step 5: Implementation of the impact of health on mortality

The dynamic structure of ATHLOS-Mic allows the use of the health module to modulate the mortality rate. In this way, changes in the risk factors also affect the number of people who survive via the health metric which is the main outcome of the health module.

Using the information on deaths between two waves available in the SHARE-HD, we estimate the impact of health on the probability of dying $q$ between $t$ and $t+a$, controlling for education, age, sex and country, with Eq. (4):

4 $$ logit(_{a} q_{t} ) = \beta_{0} + \beta_{1} \left( {a - 1} \right) + \beta_{2} HM\_gr_{t - a} + \beta_{i} X_{i, t - a} $$

Here ${\beta }_{1}$ controls for the different duration between observations and ${\beta }_{2}$ captures the effect of a categorical transformation of the health metric at time t-a. ${\beta }_{i}$ is a vector of parameters controlling the effect of socio-economics characteristics.

The original base model (CEPAM-Mic) includes population mortality rates, stratified by age, sex, and education obtained from Lutz et al.^68^. For our purposes, in ATHLOS-Mic we adjust the health coefficient ${\beta }_{2}$ in order to get contrast parameters for all health categories according to their weighted population average using the whole population as reference (see table S8 in section S3 for the contrasted parameters). Specifically, we adjust the yearly mortality rates by age, sex, country, and education that were already set in the original assumptions of the microsimulation by using the contrasted parameters (β_2_) on Eq. (5):

5 $$_{1} q_{t}^{^{\prime}} = \frac{{\exp \left( {logit(_{1} q_{t}^{{}} ) + \beta_{2} HM\_gr_{t}^{^{\prime}} } \right)}}{{1 + \exp \left( {logit(_{1} q_{t}^{{}} ) + \beta_{2} HM\_gr_{t}^{^{\prime}} } \right)}} $$

where:_1_ ${q}_{t}^{^{\prime}}$ is the age-, sex-, country, and education-specific mortality rate at time t adjusted by the health metric; _1_ ${q}_{t}$ is the age-, sex-, country, and education-specific mortality rate without adjustment.

${HM\_gr}_{t}^{^{\prime}}$ is the predicted health index (categorized) at time t.

**Figure 2:**
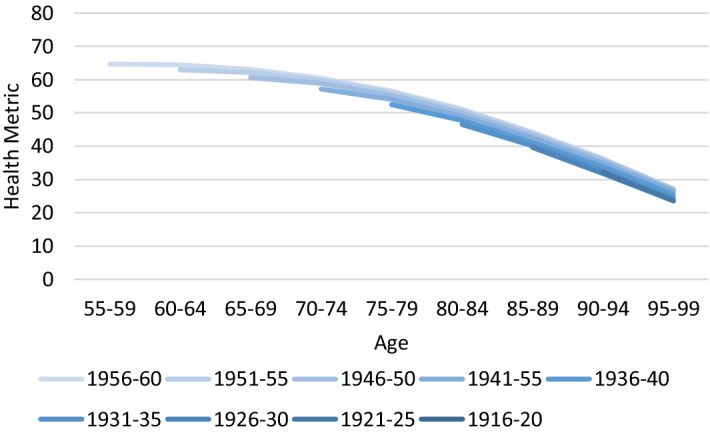
Projected average health metric by cohort over the period 2015–2060, under baseline scenario.

As these contrasted parameters were adjusted using population weighed averages, the number of deaths in the first year of the projection in ATHLOS-Mic is about the same as in CEPAM-Mic (which doesn’t include the health metric). Table S9 in the supplementary material show Person correlation coefficients and Mincer-Zarnowitz regression parameters for the number of deaths by age, sex, education, country, and year between the original projection (CEPAM-Mic) and ATHLOS-Mic. Both indicators strongly support the validity of the simulation of survivals in ATHLOS-Mic. Correlation coefficients are all above 0.9, indicating that the number of deaths by age, sex, education, country, and year are reproduced accurately. β parameters from Mincer-Zarnowitz regressions are close to 1, indicating no major systemic bias. However, slightly bigger discrepancies appear in further projected years as a result of the changing composition of the population in terms of the health metric. Since health will improve for the future elderly population (see Fig. 2 in the results section), ATHLOS-Mic outcomes result in fewer deaths rate on average. No further calibrations were done, first because the results are quite similar to the original projection, and second because, if anything, adding more sources of heterogeneity (which is the case of ATHLOS-Mic by including health in the projection) generally leads to more accurate projection outcomes^69^.

### Outcome of interests

ATHLOS-Mic can provide a large set of outcomes combining any of forecasted variables. For the purpose of this study, our analyses focus on the following three main outcomes:

- *average health,* which is the arithmetic mean of the individual health metric;
- the *number of years lived per person (NYLP)*, which is calculated by dividing to the total number of person-years by the initial population size;
- the *number of years lived per person (NYLP) in good health,* where the lower threshold of good health has been defined to a value of health metric equal to 60, given that the chances of surviving have been found to be drastically higher for the population above this threshold (see Table S8).

Those variables are also broken down by cohort, years, and level of education.

### Definition of the scenarios for the sensitivity analysis

In addition to a baseline scenario that implements all the parameters described in the sections above, we built a range of alternative scenarios to test the sensitivity of the risk factors on the projection outcomes. The scenarios are as follows:

1. *The Baseline scenario* uses all parameters from statistical models. It’s a “business-as-usual” scenario, in which cohorts are replaced through the demographic metabolism^70^. Educational attainment thus gradually improves, as older cohorts are gradually replaced by younger cohorts with higher levels of education on average.
2. *The NoAH scenario* removes the prevalence of arterial hypertension in the population;
3. *The NoObe scenario* removes the prevalence of obesity in the population;
4. *The NoSmoke scenario* removes the prevalence of smoking in the population;
5. *The NoDep scenario* removes the prevalence of depression in the population;
6. *The NoInactive scenario* assumes that everyone does physical activity;
7. The *EqEdu scenario* removes the direct effect of education inequality on the change of the health metric but keeps the effect of education on the risk factors. In other words, all things being equal, this scenario assumes that the health of individuals with a low or a medium level of education deteriorates at the same pace as those with a high level of education;
8. *The NoRisk scenario* combines scenarios 2 to 7, removing the prevalence of all risk factors and the negative effects of education inequality on the change of the health metric;
9. *The BadHMAlive scenario* eliminates the differences in mortality rate by health (i.e., the parameter for the probability of dying for people with $HM\ge 60$ is also applied to those with $HM<60.$)

Scenarios from 2 to 9 have two main purposes. First, they test how each risk factor affects ATHLOS-Mic’s outcomes. In other words, they validate the internal consistency of model regarding its different components^39^. Given that the changes in risk factors are modeled dynamically in ATHLOS-Mic, removing a risk factor, as it is assumed in scenarios 2 to 6, not only affects the health metric directly through the parameter associated to the risk factor, but also affects it indirectly by affecting the transitions of other risk factors. Second, as the comparison of their outcomes reveals which risk factor has the largest influence on the future health of the population, they provide some relevant information for policy interventions. Nevertheless, it should be stated that, clearly, none of those alternative scenarios should be interpreted as a possible or realistic future.

## Simulation results

Using ATHLOS-Mic and data from SHARE-HD, the future health trajectories of elderly individuals born before 1961 for 14 European Countries were simulated for the 9 different scenarios explained above.

### Simulation results under baseline scenario

Figure 2 presents the projected average health metric (HM) by cohort for the baseline scenario. As expected, as cohorts get older, their health deteriorates. For example, the average HM of the birth cohort of 1956 at the beginning of the simulation, specifically, at the age interval of 55–59, is 65, and declines gradually over four decades to reach an average value of 22 when the cohort reaches the age interval of 95–99. The baseline scenario also reveals that each generation will be healthier than the previous one at the same age. For example, at the age of 80–84, the cohorts born in 1956–60 will have 4 points higher health level than that in cohorts born in 1931–1935 (51 versus 47 points). Moreover, average HM of individuals born in 1956–1960 when they reach the age of 80–84 will be similar to the average HM of those who born in 1936–40 when they are at the age of 75–79. This finding also provides support for the idea in Sanderson and Scherbov (2010) that the conventional definition of the old-age threshold as a function of the number of years since birth should be revised, as it doesn’t account for better health and increasing life expectancy.

For a better understanding of the improvement in the health of the later-born cohorts presented in Fig. 2, we also projected the prevalence of risk factors by cohort and age (see figure S3) to find that younger cohorts tend to have better health behaviors and less adverse health conditions than older cohorts. In fact, compared to older cohorts at the same age, in younger cohorts, the prevalence of arterial hypertension, depression, obesity, and smoking is lower, while the level of physical activity is higher. However, these differences are quite small. For instance, the prevalence of arterial hypertension at the age of 80–84 is 46 percent for the cohort 1931–1935, while it is projected to be 43 percent for the cohort 1956–60. Similarly, the difference in the prevalence of obesity at the same age interval is only 2 percent between these two birth cohorts (28% versus 30%). Overall, a consistent improvement is observed over cohorts and for all age, but it is not found to be very significant.

However, trends are very different by education, as the educational composition of the population also changes over time. Beyond a certain age, the level of education doesn’t change much and becomes a persistent characteristic, therefore only the change in the proportion of different education levels in a cohort can be attributable to the differences in mortality rates. The improvement in the levels of educational attainment over cohorts is found to be both consistent and significant (see Figure S4). In particular, 70% of the people born before 1930 have low levels of education, this ratio decreases for subsequent cohorts, and passes below 30% for 1956–1960 generations. Since the level of education has been found the most important factor affecting both the health score and the risk factors, the observed increase in the levels of education over time is expected to improve the health of the younger cohorts. Indeed, the projected education-specific levels of the health metric are not expected to change significantly over time, what changes is the educational composition of later born cohorts, making them comparably healthier.

**Figure 3:**
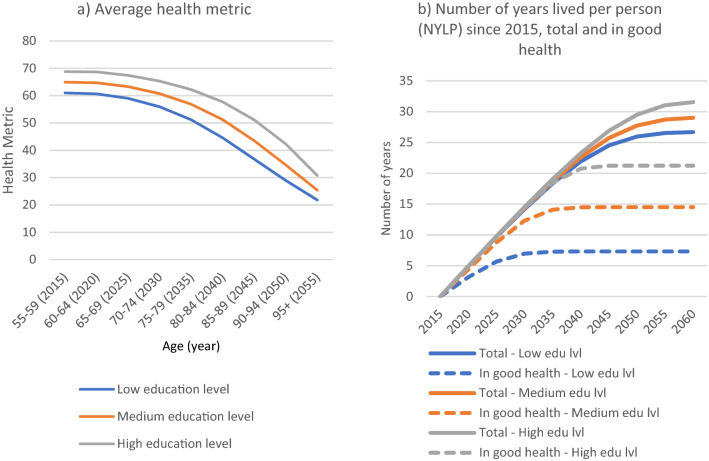
Projected average health metric and NYLP by education level for the cohort 1956–1960 under the baseline scenario.

Since the level of education has a big impact on the health metric and also plays a major role in the prevalence of risk factors, we show in Fig. 3 the health trajectories by education level for the cohort of 1956–60, in order to visually bring out inequalities in health by education within the same cohort. The education gap in the average health metric is quite obvious (Fig. 3a), as the average health score of the people with high levels of education is already 10 points higher than the health score of the people with low levels of education (69 versus 61 points) at the beginning of the projection period (at the age of 55–59). Moreover, the difference in health by education remains almost constant throughout the projection period, except in 2050 (at the age of 90–94) when it reaches to 13 points (42 versus 29). Indeed, this gap can roughly be translated into a 10 years difference, as the health score of someone with high levels of education at the age of 80–84 will be about the same as someone with a low level of education at the age of 70–74 (58 vs 56 points).

As the health metric also affects the mortality rates, we projected the number of years lived per person (NYLP) over the period 2015–2060 and the NYLP lived in good health (see Fig. 3b). The analysis of the parameters reveals that the probability of dying starts markedly increasing when the value of HM is below 60. Therefore, we used this value as the threshold to create a proxy indicator for having a good health, and by extension, to calculate the number of years lived per person in good health both for average and by education level.

Looking at the projected NYLPs by education shown in Fig. 3b, the advantages of having a high level of education can be clearly seen. Indeed, the cohort born between 1956 and 1960 can expect to live 32 years more at the age of 55–59 if they have high levels of education. For those with low levels of education, life expectancy is only 27 years. Furthermore, the gap is bigger for the NYLP in good health (i.e., HM >  = 60), namely, 21 years for people with a high level of education compared to only 7 years for those with a low level of education. In other words, at the age of 55–60, people with a high level of education would live almost 70% of their remaining life in good health, while this proportion would only be of 27% for those with a low level of education. Overall, education matters not only for the number of years lived, but also for the quality of life in these years.

#### Comparison of simulation results under different scenarios

**Figure 4:**
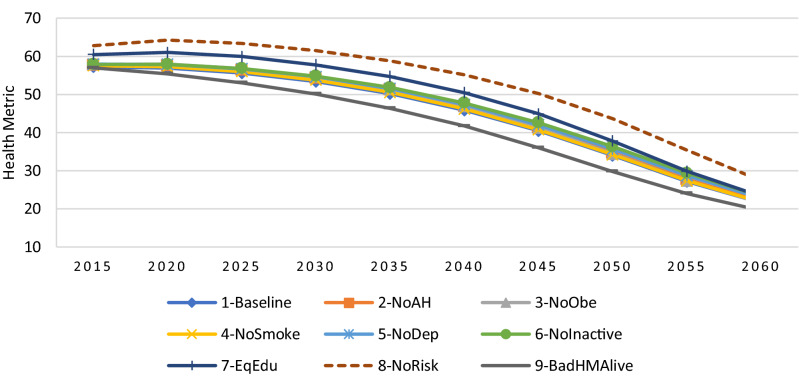
Projected average health metric for cohorts 1916–1960 under different scenarios.

In Fig. 4, we present the projected average health metric for cohorts born between 1916 and 1960 according to the 9 different scenarios described previously. When all risk factors are removed, the HM is improved significantly. In the baseline scenario, as the population ages, the average HM which is 57 in 2015, declines steadily throughout the projected period and reaches to 22 at the end of the period in 2060. In the scenario 8-NoRisk, the average HM is consistently higher than the average HM in other scenarios (approximately 5 or 6 points) over the projected period 2015–2060. Among those scenarios, the one which has the strongest effect on health is the scenario in which the inequalities in education are eliminated (i.e., EqEdu). On the other hand, the scenario BadHMAlive, that eliminates the differences in mortality rate by health, has the worst effect on the average HM, since it keeps the people in bad health alive for a longer time.

**Figure 5:**
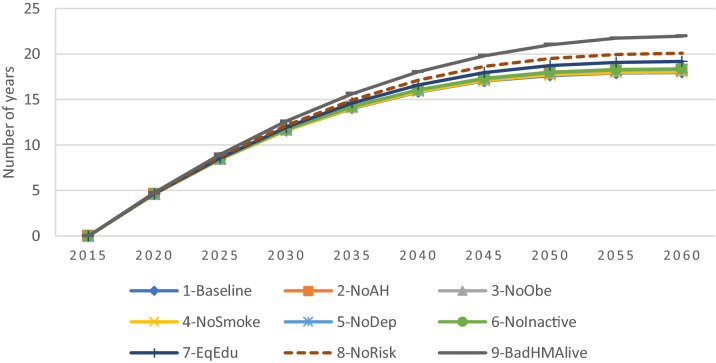
Projected number of years lived per person (NYLP) from 2015 to 2060 under different scenarios.

Figure 5, shows that the average number of years lived per person under all scenarios. In the baseline scenario, the projected NYLP reaches 18 by 2060, indicating that the projected population would have lived 18 years on average until 2060. On the other hand, looking at alternative scenarios, we see that removing all risk factors (i.e., scenario NoRisk) increases the average NYLP by around 2 years in 2060, compared to the baseline scenario. Similarly, the projected NYLP under the BadHMAlive scenario (that removes the difference in death rates between people in good and bad health) is 4 years higher than the projected NYLP in the baseline scenario in 2060. However, as discussed previously, the average health of the population would also be lower under the BADHMAlive scenario. Nonetheless, we can see that the scenario which assumes the same speed of change in health over time regardless of the levels of education (i.e., EqEdu) has the most significant effect on the NYLP, as it increases the projected NYLP by around 1.2 years, whereas the scenarios that remove other risk factors slightly improve the NYLP by less than 0.5 years.

**Figure 6:**
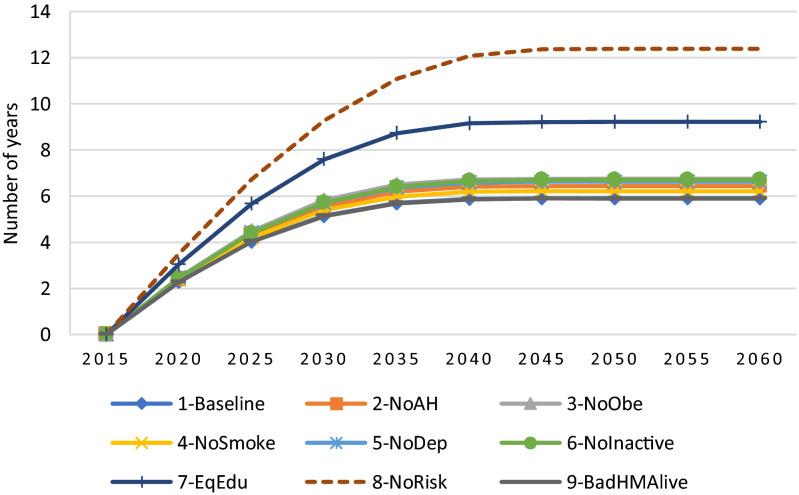
Projected Number of years lived per person (NYLP) in good health ($HM\ge$ 60) from 2015 to 2060 under different scenarios.

Looking at the NYLP in good health ($HM\ge$ 60) shown in Fig. 6, we can clearly see large variations between the alternative scenarios. For example, under the NoRisk scenario (which removes all risk factors) the NYLP in good health more than doubled and would exceed 12 years by 2060. Similarly, the scenario removing inequalities in education (i.e., EqEdu) is the one having the largest impact on the NYLP in good health, as it reaches 9.2 years by 2060, a gain of more than 3 years compared to the baseline scenario. The gains in NYPL in good health under the remaining scenarios with respect to the baseline scenario vary between 0.4 to 2 years. Furthermore, the difference between the baseline scenario and the other scenarios is much lower, between 0 and 1 year, and is particularly small under the NoSmoke scenario (with a gain of only 0.3 by 2060).

## Discussion and conclusion

In this study, we first present the design of a dynamic microsimulation model, ATHLOS-Mic, that has been developed for purpose of projecting the future health trajectories of individuals using a set of risk factors among other demographic characteristics. Here, we used the model to project the health trajectories of elderly population aged 65 years and older from 2015 to 2060 for 14 European Countries under different scenarios. We also provide first estimates of the average number of years lived per person as well as the average number of years lived in good health in the prevalence of different risk factors. Different from the related literature, we use a novel composite health metric which has ability to summarize multiple aspects of health related to physical and cognitive functioning in a single continuous variable.

This study links the literature focusing on ageing and health with the literature on population projections in two ways. First, it provides a population projection framework which is well-suited for the study and projection of population health-aging trajectories, taking individual-level heterogeneity, and the dynamic relationships among risk factors, health, and mortality into consideration. Second, by using a novel metric defining health on the basis of functional ability, which has a central role in the planning of policies for elderly populations, as well as in health research and welfare analysis, as functionality is closely related to the level of well-being in older ages.

From a methodological point of view, ATHLOS-Mic, benefiting from the advantages of dynamic microsimulation modeling framework, improves the overall quality of population projections by allowing the incorporation of health into population projections, and by including more sources of heterogeneity beyond age and sex within population groups, than the standard demographic population models are able to provide. Given the importance of knowledge on future health trajectories of older people for a vast variety of planning activities and policies, the outputs generated by ATHLOS-Mic provide relevant information to policy makers about population ageing and potential health profiles.

Moreover, this framework provides a very flexible tool, as it can measure the effect of changes along several dimensions and allowing for a wide array of “what if” scenarios. In this paper, we designed 8 scenarios for testing interactions between risk factors, health, and mortality. Given the methodological scope of this study, these scenarios have been designed to serve as a validation tool used in testing for the internal consistency of our model rather than a realistic policy tool aiming to help policy makers to choose appropriate actions to improve health and welfare. More realistic and policy-oriented scenarios will be developed in further studies, however, the outcomes of these scenarios still provide relevant information about the relationship between health and risk factors, as they can be used to rank the risk factors based on their expected impact on the future health status of the elderly population. However, given the large heterogeneity of the 14 European countries – with different social and health systems, these outcomes should be taken with caution.

Our scenario analysis revealed that the educational attainment stands out among other risk factors as the main source of inequality in health. In fact, the scenario that removes the effect of having a low level of education on the health metric is the one having the largest effect on the projected average health metric (increased by 3 to 5 points compared to the baseline scenario), the average number of years lived per person (increased by 1.2 years compared to the baseline scenario), and the average number of years lived in good health (increased by more than 3 years). There are two dimensions that explain this big effect. First, the parameter associated to a low level of education (and to a less extend to medium education) in the regression modeling the health metric is much larger than those for other risk factors, implying that, on average, at an individual level, having a low level of education has a major effect on health. Second, the share of those with low levels of education in the population (about 45% in 2015) is the large and, in consequence, a sizable number of individuals would be affected by a change in the size of the effect of this component. On the other hand, it may be surprising that the scenario which removes the effect of inequalities in education on health has larger impact than the scenario that changes the prevalence of risk factors whose effects on health are widely acknowledged, such as smoking or physical activity. This may occur due to possible measurement issues that bias their statistical association with health, as our statistical models can consider the impact of these risk factors on health to a limited extent given that we have observations for these variables for only a few years.

Nevertheless, the most likely explanation for this result is that education has a series of cascading effects on health. A large body of the literature has analyzed educational attainment as a fundamental cause of health^71,72^, however, the channels through which education influences health are found to be largely complex and not clearly defined^73^. Previous studies have indicated that education affects health not only directly but also indirectly through a set of mediators including socio-economic status, psychological and interpersonal resources, and health behaviors and health care utilization^74–76^. Moreover, the effect of these mediators is not the same and they may vary across the life course. Overall, these studies coincide in the fact that relationship between education and health should be examined not only across the life course but also across time^77^.

Moreover, coming back to the relatively small effect of other risk factors, since people who experienced a deterioration in their health are probably more likely to stop smoking or doing physical activity, the causal relation between health and smoking could be reversed (i.e. smoking causes bad health, but bad health causes stop smoking). Indeed, using a similar health metric, Caballero et al.^36^ found out that former smokers have worse health than current smokers and, similarly, that people in bad health are already more likely to not drink. Even though in ATHLOS-Mic we model changes in the health metric, the observation period in the estimation sample (2 years on average) is probably too short to completely remove these reversed causal relationships and to properly measure the long-term effect of bad life habits on the health deterioration. We also suspect similar issues for obesity, as people who are in bad conditions might dramatically lose weight and have a very low body mass index^78^. A multinomial variable distinguishing underweight people would thus be more appropriate to measure the net effect of obesity on the deterioration of health. Nevertheless, having more information on risk factors during adult life should improve the modeling of the health metric, as the effect of bad life habits take several years to disappear. This could also partly explain the strong effect of education in our model, as the effect of past bad life habits could be reflected on it.

Overall, our projections highlight the central importance of education, and quantifies the effects of the risk factors on the future of health trajectories in Europe. In the future, although young cohorts are likely to be healthier than their previous cohorts on average, the number of years they will spend in good health largely depends on the dynamics of these risk factors. Given that ill health may increase the need for care and health services, and therefore, health expenditures, quantifying the possible impact of each risk factor on the average number of years in good health on the next 40 years is crucial for policy makers as it may help in prioritizing the prevention of risk factors, while designing health policies aiming for healthy aging. Given the paramount importance of educational attainment for health, future research will use the ATHLOS-Mic to generate more realistic policy scenarios to explore the role of different education policy options in reducing or eliminating the existing and growing disparities in health and mortality, in order to help shaping policy decisions.

## Supplementary Information

Supplementary Information.

## Data Availability

The datasets generated during and analyzed during the current study are not publicly available for confidential reasons. Aggregate results are available from the corresponding author on reasonable request.
