# Supplementary material for: Projecting health-ageing trajectories in Europe using a dynamic microsimulation model
Source: Sci Rep. 2021 Jan 19;11:1785. doi: 10.1038/s41598-021-81092-z (PMC7815779; doi:10.1038/s41598-021-81092-z)
Supplement: Supplementary file 1 — Supplementary Information. [file 41598_2021_81092_MOESM1_ESM.docx]

**Projecting Health-Ageing Trajectories in Europe Using a Dynamic Microsimulation Model**

**Supplementary Materials**

Guillaume Marois^a, b^ *, Arda Aktas^b^

^a^ Asian Demographic Research Institute, School of Sociology and Political Sciences, Shanghai University, 99 Shangda Rd., Shanghai, 200444, China

^b^ Wittgenstein Centre for Demography and Global Human Capital (University of Vienna, IIASA, VID/ÖAW), International Institute for Applied Systems Analysis, 2361 Laxenburg, Austria

*Corresponding Author

Email: [marois@iiasa.ac.at](mailto:marois@iiasa.ac.at)

**S.1. Measurement of Health**

In our analysis, as a measure of health of individuals, we use a composite index of health that has been developed as a part of the ATHLOS project. The health metric and its methodology have been already presented and validated, first in Caballero et al.^1^, as well as in other studies (e.g., De la Fuente et al.^2^; Daskalopopoulou et al.^3^) using data sets such as the English Longitudinal Study of Ageing (ELSA), the Health and Retirement Study (HRS) and the Mexican Health and Aging Study (MHAS) which were designed under the sample principles as the SHARE dataset used in our study. The construction of the health metric is based on the assumption that there exists a latent measure of the health of individuals in a sample that can be inferred from a set of observed health-related characteristics. More specifically, using a Bayesian multilevel item-response theory (IRT) approach and a set of health characteristics, including self-reported health questions and measured tests obtained from the longitudinal household surveys, the distribution of a health score is estimated in a way that it reflects the distribution of the observed health status (represented by the health-related characteristics) of a particular sample. Posteriorly, the uni-dimensionality of health variables used in the estimation of the metric are assessed by, first, conducting an Exploratory Factor Analysis (EFA) to detect the latent structure among the items and, second, applying a Confirmatory Factor Analysis (CFA) to gather evident for creating a global health score. To confirm the predictive validity of the health metric, in Caballero et al.^1^, a Receiver Operating Characteristics (ROC) curve analysis is performed adjusted per gender, where mortality was assumed as the gold-standard measure to examine the associations of the baseline metric with mortality observed over increasing periods of time (such as 2, 4, and 10 years follow-up period) by calculating the Area Under the ROC Curve (AUC). Other similar analyses were performed by looking at institutionalization rates, as well as by assessing the relationship between the presence of chronic conditions and the health score using a multiple linear regression model. As for the suitability of the health metric for causal inference, there are several studies, some published and some in preparation, where this has been examined^4,5^.

**S.2. Data**

**S.2.1. Limitations of SHARE**

Although SHARE is one of the most comprehensive survey for the older population in Europe, it still suffers from some data limitations which restrict the projection possibilities:

- First, only the individuals aged 50 or older are surveyed. This limits the projected of population to these cohorts, because no information on health nor the risk factors is available for younger cohorts.
- Second, it does not cover all the countries in the European Union: only 18 countries have participated in at least one wave. Moreover, the participation to at least 2 waves is necessary to have longitudinal data allowing the estimation of transition rates. Only 14 countries meet this requirement, which are showed in table 1 with their respective samples size.
- Third, even for countries participating in more than one wave, the observed attrition rate is high, as it is the case in any longitudinal survey. (see Table 1)
- Finally, to be included in a transition estimation model, individuals need to have non-missing values for the variables of interest, as well as for the covariates in two consecutive waves. Since a sizable proportion of missing values is observed in at least one of these variables, the usable sample size for the modelling is reduced.

**S.2.2. The Choice of Risk Factors**

The risk factors used in the model are chosen based on empirical findings on various risk factors and their effect on health, as well as data availability.

Given these criteria, a variable measuring alcohol consumption was excluded from the module because both a previous study ^1^ and our preliminary analysis showed a positive association with the health metric. Indeed, previous studies proved that the relationship between alcohol consumption and mortality has a J-shape ^6^. Since the variable available in SHARE-HD cannot properly distinguish an alcoholic from a moderate drinker, it fails to appropriately capture the negative effect of alcoholism on health. Moreover, some studies suggest that people in bad health tend to reduce their alcohol consumption, thus reversing the causal effect ^7^.

On the other hand, although smoking is considered as one of the risk factors, it is particularly important to distinguish past smokers from those who never smoked as, after quitting smoking, it may still take many years for individuals to reach the same mortality risk of those who has never smoked ^8^. However, this is not possible in our sample as the proportion of the missing values for the variable “smoking”, which could allow us to make a distinction among current smoker, past smokers and those who have never smoked, is too high to conduct a robust statistical analysis (36% is missing in four waves). Therefore, we used a binary variable instead, where the proportion of missing values is at a reasonably low level (3%).

**S.3. Methodology: Construction of Athlos-Mic**

The ATHLOS-Mic modelled on CEPAM-Mic in several steps. This section provides a more detailed description of some of these steps, as well as the related tables presenting the estimation results from the models explained in the main text.

The ATHLOS-Mic model adds these 5 additional steps into CEPAM-Mic:

**Step 1:** Imputation of initial health and risk factors to the base population

Table S1. Correlation coefficients (Pearson) between the base population and the source data in the proportion of each imputed variable by age, sex, country, and education

| **Age group** | **Imputed variable** | | | | | |
| --- | --- | --- | --- | --- | --- | --- |
|  | **Arterial hypertension** | **Depression** | **Obesity** | **Smoking** | **Physical activity** | **Health Metric** |
| 50-54 | 0.983 | 0.973 | 0.987 | 0.979 | 0.980 | 0.971 |
| 55-59 | 0.978 | 0.980 | 0.988 | 0.983 | 0.982 | 0.970 |
| 60-64 | 0.981 | 0.989 | 0.989 | 0.987 | 0.984 | 0.970 |
| 65-69 | 0.990 | 0.985 | 0.982 | 0.992 | 0.989 | 0.965 |
| 70-74 | 0.989 | 0.990 | 0.992 | 0.986 | 0.989 | 0.971 |
| 75-79 | 0.982 | 0.978 | 0.985 | 0.977 | 0.976 | 0.963 |
| 80+ | 0.983 | 0.988 | 0.984 | 0.979 | 0.980 | 0.949 |
| **Total** | **0.984** | **0.984** | **0.987** | **0.984** | **0.983** | **0.967** |

Table S2. Correlation coefficients (Pearson) between the base population and the source data in the proportion of each imputed variable by other imputed variables

| **Health metric group** | **Imputed variable** | | | | | |
| --- | --- | --- | --- | --- | --- | --- |
|  | **Arterial hypertension** | **Depression** | **Obesity** | **Smoking** | **Physical activity** | **Health Metric** |
| [0-30[ | 0.919 | 0.969 | 0.943 | 0.971 | 0.944 | 0.965 |
| [30-40[ | 0.992 | 0.995 | 0.989 | 0.987 | 0.991 | 0.989 |
| [40-50[ | 0.994 | 0.995 | 0.994 | 0.995 | 0.993 | 0.988 |
| [50-60[ | 0.995 | 0.997 | 0.997 | 0.998 | 0.985 | 0.989 |
| [60,100] | 0.995 | 0.997 | 0.994 | 0.997 | 0.996 | 0.996 |
| **Total** | **0.982** | **0.991** | **0.985** | **0.989** | **0.982** | **0.989** |

We also performed Mincer-Zarnowitz regressions on the logarithm of the prevalence of each imputed variables, broken down by age, sex, country and education and by other imputed variables. This procedure can be used to compare two sets of data describing the same variables, such as observed and imputed values of population counts^10^. In our case, regressions predict the logarithm of the population count from the source dataset (SHARE-HD 2011) by the logarithm of the population counts from the imputed base-population (I):

$ln(S)=\propto+\beta*ln(I)$ (Eq.S1)

A parameter β that is statistically different from 1 indicates a systematic *relative* difference between the prevalence of the risk factors in the two datasets affecting all subgroups. Table SX and SX present parameters.

Table S3. Value of β parameters from Mincer-Zarnowitz regressions

|  | | **Population broken down** | |
| --- | --- | --- | --- |
| **Imputed variables** | | **by age/sex/**  **edu/country** | **by other imputed variables** |
| Arterial hypertension | | 0.998 (0.001) | 1.000 (0.002) |
| Depression | | 0.998 (0.001) | 0.999 (0.001) |
| Obesity | | 0.994 (0.002) | 0.991 (0.003) |
| Smoking | | 1.003 (0.002) | 0.997 (0.004) |
| Physical activity | | 0.998 (0.001) | 0.997 (0.004) |
| Health metric group | [0-30[ | 1.002 (0.007) | 0.950 (0.013) |
|  | [30-40[ | 0.999 (0.004) | 0.982 (0.005) |
|  | [40-50[ | 0.993 (0.002) | 0.994 (0.002) |
|  | [50-60[ | 0.998 (0.002) | 1.004 (0.002) |
|  | [60,100] | 0.998 (0.001) | 1.002 (0.002) |

**Step 2:** Modeling the changes in sociodemographic characteristics

**Step 3:** Modelling the changes in behavioral and bio-medical risk factors

Table S4.: Estimation results for 5 risk factors

| **Covariate (t-1)** | | **Model I** | | **Model II** | | **Model III** | | **Model IV** | | **Model V** | | |
| --- | --- | --- | --- | --- | --- | --- | --- | --- | --- | --- | --- | --- |
|  |  | **Smoking (t)** | | **Depression (t)** | | **Physical**  **activity (t)** | | **Obesity (t)** | | **Arterial**  **hypertension (t)** | | |
| Intercept | | 1.031 |  | 0.987 |  | -2.622 | ** | -4.371 | ** | -8.435 | *** | |
| Age | | -0.092 |  | -0.110 | *** | 0.144 | *** | 0.075 | * | 0.189 | *** | |
| Age*Age | | 0.000 |  | -0.000 | *** | -0.001 | *** | -0.000 | ** | -0.001 | *** | |
| Sex=F | | -0.101 |  | 0.491 | *** | -0.242 | *** | -0.003 |  | 0.012 |  | |
| Edu=Low | | 0.007 |  | 0.493 | *** | -0.244 | *** | 0.692 | *** | 0.313 | *** | |
| Edu=Med | | 0.094 |  | 0.235 | *** | -0.160 | ** | 0.358 | *** | 0.182 | ** | |
| Smoking | | 4.288 | *** | 0.197 | ** | -0.261 | *** | -0.068 |  | -0.041 |  | |
| Depression | | -0.040 |  | 1.665 | *** | -0.399 | *** | 0.028 |  | 0.052 |  | |
| Physical activity | | -0.058 |  | -0.234 | *** | 1.267 | *** | -0.124 | * | -0.082 | * | |
| Obesity | | -0.033 |  | 0.125 | * | -0.333 | *** | 3.853 | *** | 0.513 | *** | |
| Arterial hypertension | | -0.005 |  | 0.110 | ** | -0.140 | ** | 0.345 | *** | 2.625 | *** | |
| Country (ref=DE) | |  |  |  |  |  |  |  |  |  |  | |
|  | AT | -0.019 |  | -0.232 | ** | -0.113 |  | -0.157 |  | -0.171 | * | |
|  | BE | -0.079 |  | 0.050 |  | -0.490 | *** | -0.311 | ** | -0.352 | *** | |
|  | CZ | 0.330 | ** | -0.022 |  | -0.434 | *** | -0.022 |  | 0.050 |  | |
|  | DK | -0.236 | * | -0.180 | * | -0.176 | ** | -0.259 | ** | -0.294 | *** | |
|  | EE | 0.057 |  | 0.320 | *** | 0.119 |  | -0.005 |  | -0.034 |  | |
|  | ES | -0.370 | ** | -0.045 |  | -0.588 | *** | -0.324 | ** | -0.334 | *** | |
|  | FR | -0.177 |  | 0.259 | ** | -0.427 | *** | -0.411 | *** | -0.462 | *** | |
|  | GR | 0.362 | ** | -0.673 | *** | 0.644 | *** | -0.463 | *** | -0.398 | *** | |
|  | IT | -0.157 |  | 0.193 | * | -0.586 | *** | -0.721 | *** | -0.263 | ** | |
|  | NL | -0.180 |  | -0.427 | *** | 0.125 |  | -0.309 | ** | -0.510 | *** | |
|  | PL | -0.156 |  | 0.103 |  | -0.436 | *** | 0.021 |  | -0.410 | ** | |
|  | SE | -0.316 | * | -0.290 | ** | 0.074 |  | -0.374 | ** | -0.223 | ** | |
|  | SI | -0.049 |  | 0.038 |  | 0.119 |  | 0.002 |  | 0.071 |  | |
| Duration | | -0.006 |  | 0.082 | *** | -0.057 | ** | -0.045 |  | 0.088 | *** | |
| **Note:** P values: *p<0.5; **p<0.01; ***p<0.0001 | | | | | | | | | | | |  |
| **Source:** Authors' estimation using data from SHARE-HD and the methods of Generalized Estimating Equations (GEEs)  Table S5 Association of Predicted Probabilities and Observed Responses   \| **Model** \| **Percent concordant** \| **C-Statistic** \| \| --- \| --- \| --- \| \| I-Smoke \| 0.889 \| 0.893 \| \| II-Depression \| 0.745 \| 0.747 \| \| III-Physical activity \| 0.753 \| 0.754 \| \| IV-Obesity \| 0.857 \| 0.860 \| \| V-Arterial hypertension \| 0.822 \| 0.824 \|   The estimated coefficients are used to derive the net probability of yearly changes in the risk factors for a standardized profile of an individual living in one of the EU countries in our sample. For example, using the coefficient of the parameters in eq. 1, we calculate this probably for a 65 years old individual from Germany who smoked in the previous period as 77.8% (i.e., exp(1.031+4.288+65*-0.092+65^2^*4.549E-04)/(1+exp(1.031+4.288+65*-0.092+65^2^*4.549E-04))), implying that a 65 years old from Germany who smoked last year will keep smoking with a 77.8% chance when s/he turns into 66 years old (all other thing being equal), or conversely, s/he will quit smoking with a 22.2% chance (100%-77.8%). On the other hand, the probability of smoking at time $t$is only for 4.6% (i.e., exp(1.031+65*-0.092+652*4.549E-04)/(1+exp(1.031+65*-0.092+652*4.549E-04))) for this individual, if s/he did not smoke previous period. In this way, using the estimated coefficients presented in Table S1, we calculated the net transition rates (from time $t-1$to $t$) for all risk factors and individual characteristics. | | | | | | | | | | | |  |

Table S6: Net transition rates for risk factors, Germany

| **Risk factor_t_** | **Risk factor_t-1_=1** | **Risk factor_t-1_=0** |
| --- | --- | --- |
| **Smoker** | 22.2% | 4.6% |
| **Depression** | 66.2% | 8.8% |
| **Physical activity** | 14.7% | 62.1% |
| **Obesity** | 24.1% | 6.3% |
| **Arterial hypertension** | 25.6% | 17.4% |
| **Source:** Authors' calculation using the estimated parameters presented in table S1 | | |

| Figure S1: Age pattern for risk factors (net effect from other covariates) |
| --- |
|  |
|  |

Source: Authors’ calculation using the estimated parameters from table

Using the initial values of the risk factors imputed to the base population $F_{t}^{'}$in step (ii) and the estimated parameters, Monte Carlo simulations are performed to predict stochastically the value of the risk factors at time $t+1$ throughout the projection period. It can be defined mathematically with the following formula presented by Eq.S1:

$F_{t+1}=\frac{\exp\left( \beta_{0} {+ \beta}_{2}F_{t}^{'}+\beta_{i}X_{i, t} \right)}{1+\exp\left( \beta_{0} {+ \beta}_{2}F_{t}^{'}+\beta_{i}X_{i, t} \right)}$

$F_{t+1}^{'}\left\{ \begin{aligned} 1 F_{t+1}<Z\sim\left( \left[ 0,1 \right] \right) \\ 0 F_{t+1}\geq Z\sim\left( \left[ 0,1 \right] \right) \end{aligned} \right.$ (Eq. S2)

where $Z\sim\left( \left[ 0,1 \right] \right)$ is a random number uniformly distributed between 0 and 1.

**Step 4:** Modeling the change in the health

Table S7: Estimation results for the model of change in the health

| **Covariate (t-1)** | | **Parameter** | **p-value** |
| --- | --- | --- | --- |
| Intercept | | 0.694 | *** |
| HM | | -4.444 | *** |
| HM*HM | | 8.739 | *** |
| HM*HM*HM | | -6.214 | *** |
| Age | | 1.20E-02 | *** |
| Age*Age | | -1.30E-04 | *** |
| Sex=F | | -0.015 | *** |
| Edu=Low | | -0.059 | *** |
| Edu=Med | | -0.033 | *** |
| Smoking | | -0.020 | *** |
| Depression | | -0.035 | *** |
| Physical activity | | 0.017 | *** |
| Obesity | | -0.033 | *** |
| Arterial hypertension | | -0.011 | ** |
| Country of residence (ref=DE) | |  |  |
|  | AT | 0.023 | ** |
|  | BE | 0.005 |  |
|  | CZ | 0.002 |  |
|  | DK | 0.036 | *** |
|  | EE | -0.041 | *** |
|  | ES | 0.009 |  |
|  | FR | 0.004 |  |
|  | GR | 0.013 | * |
|  | IT | -0.006 |  |
|  | NL | 0.032 | *** |
|  | PL | 0.015 | * |
|  | SE | 0.037 | *** |
|  | SI | 0.017 | * |
| **Note:** P values: *p<0.5; **p<0.01; ***p<0.0001 | | | |
| **Source:** Authors' estimation using data from SHARE-HD and the method of Generalized Estimating Equations (GEE) | | | |

The results show that smoking (-0.020), depression (-0.035), obesity (-0.033) and arterial hypertension (-0.011) accelerate the decline in health, while physical activity (0.017) slows it down. On the other hand, age-related changes in health follow a quadratic trend, with steeper declines at older ages. Nevertheless, the depreciation rate in health observed for women is higher than men. The level of educational attainment is found to be a major factor affecting the change in the health metric substantially. In fact, the health tends to decline faster for people with low education (-0.059) or medium education (-0.033) compared to those with high education. Finally, the results reveal some national variations in the rate of the decline in health. For example, all other things being equal, the health declines faster in Estonia (-0.041) and but slower in Denmark (0.036) compared to Germany. Overall, the estimated parameters are similar to those estimated in Caballero et al. ^1^, assessing the effects of covariates on a similar health metric that had been constructed using the data from the English Longitudinal Study of Ageing.

Using the parameters from Table S7 and observed values in our sample, Figure S2 compares the predicted and observed changes in the logit of the HM over one year for Germany. As seen in the figure, people, who were initially in good health, are more likely to experience a decline in health while their peers, who were initially in bad health, are likely to see their health improving if they survive. For those who were in average health, almost no change in their health is observed.

| Figure S2 – Predicted and observed change in the logit of the HM between t and t+1 based on the value of the HM at time t-1 |
| --- |
|  |

Source: Authors’ calculation using the estimated parameters from table S3 and observed values in the sample.

**Step 5:** Implementation of the impact of the health on mortality

Table S8: Contrasted parameters for β2 from eq. 5

| **Health metric** | **Parameter** | **Odd ratios** | **Std. Err.** | **% of N** |
| --- | --- | --- | --- | --- |
| **[0, 30[** | 1.946 | 6.999 | 0.199 | 4.1% |
| **[30, 40[** | 1.235 | 3.437 | 0.165 | 7.4% |
| **[40, 50[** | 0.438 | 1.550 | 0.120 | 16.0% |
| **[50, 60[** | 0.019 | 1.019 | 0.103 | 23.1% |
| **[60, 100]** | -0.457 | 0.633 | 0.079 | 49.3% |
| **Percent Concordant** | | 0.830 | | |
| **C-Statistic** | | 0.837 | | |

As shown in Table S8, individuals with a HM above 60 or with a HM below 30 have been grouped into single categories, as little differences were observed in the mortality rates of the individuals in these respective categories. Note that about 70% of the population has an HM above 50, and that, above this threshold, the observed differences in mortality rates are quite low. For instance, the odds ratio for the category [60,100] is 0.633, which indicates that the probability of dying for individuals in this group is only 34% lower than the one of the average individual of the same age, sex, country, and education. On the opposite side, the parameter for the category [0, 30[ corresponds to an odds ratio of 6.999, meaning that the probability of dying for those individuals is about 7 times higher than the average. However, only 4% of the population belongs to this category. Both the C-Statistic and the percent of concordance of the model are above 0.8, validating its strong predictive power.

To validate the survival of individuals in ATHLOS-Mic, we calculated Person correlation coefficients in the number of deaths by age, sex, education, country and year between ATHLOS-Mic and the original projection model (CEPAM-Mic). We also performed Mincer- Zarnowitz regressions^10^ to test systemic biases, as expressed by equation S3.

$ln(D^{CEPAM-Mic})=\propto+\beta*ln(D^{ATHLOS-Mic})$ (Eq. S3)

Results are reported in table S9. Since parameters slightly above 1, there is a small systemic bias indicating fewer deaths in ATHLOS-Mic, which is caused by the inclusion of health in the projection as source of heterogeneity. As health is projected to improve over years, mortality rates are slightly lower, in particular at the end of the projection.

Table S9. Validation of the simulation of deaths (number of deaths by age, sex, education, country and year, ATHLOS-Mic vs CEPAM-Mic)

| **Period** | **Person correlation coefficients** | **β parameter from Mincer-** **Zarnowitz Regression** |
| --- | --- | --- |
| 2015-2019 | 0.960 | 1.012 (0.002) |
| 2020-2024 | 0.921 | 1.016 (0.003) |
| 2025-2029 | 0.929 | 1.019 (0.003) |
| 2030-2034 | 0.945 | 1.015 (0.002) |
| 2035-2039 | 0.941 | 1.013 (0.003) |
| 2040-2044 | 0.942 | 1.014 (0.003) |
| 2045-2049 | 0.951 | 1.023 (0.003) |
| 2050-2054 | 0.958 | 1.051 (0.004) |
| 2055-2059 | 0.908 | 1.105 (0.011) |
| Total | 0.938 | 1.019 (0.001) |

**S.4. Simulation Results:**

| Figure S3 Projected risk factors by age and cohorts, under baseline scenario, over the period 2015-2060 | |
| --- | --- |
|  |  |
|  |  |
|  |  |

Figure S4: Proportion of the population with low education by age

Figure S5: Projected Number of years lived per person (NYLP) since 2015, total and in good health, under baseline scenario

**References**

1. Caballero, F. F. *et al.* Advanced analytical methodologies for measuring healthy ageing and its determinants, using factor analysis and machine learning techniques: the ATHLOS project. *Scientific Reports* **7**, 43955 (2017).

2. de la Fuente, J. *et al.* Determinants of Health Trajectories in England and the United States: An Approach to Identify Different Patterns of Healthy Aging. *The Journals of Gerontology: Series A* **73**, 1512–1518 (2018).

3. Daskalopoulou, C. *et al.* Healthy ageing trajectories and lifestyle behaviour: the Mexican Health and Aging Study. *Scientific Reports* **9**, 11041 (2019).

4. Kollia, N. *et al.* Social determinants, health status and 10-year mortality among 10,906 older adults from the English longitudinal study of aging: the ATHLOS project. *BMC Public Health* **18**, 1357 (2018).

5. Wu, J. T., Leung, K. & Leung, G. M. Nowcasting and forecasting the potential domestic and international spread of the 2019-nCoV outbreak originating in Wuhan, China: a modelling study. *The Lancet* **395**, 689–697 (2020).

6. Ferreira, M. P. & Weems, M. K. S. Alcohol Consumption by Aging Adults in the United States: Health Benefits and Detriments. *Journal of the American Dietetic Association* **108**, 1668–1676 (2008).

7. Holdsworth, C. *et al.* Is regular drinking in later life an indicator of good health? Evidence from the English Longitudinal Study of Ageing. *J Epidemiol Community Health* **70**, 764 (2016).

8. U.S. Department of Health and Human Services. *The health consequences of smoking: A report of the surgeon general*. (2004).

9. Mincer, J. A. & Zarnowitz, V. The Evaluation of Economic Forecasts. in *NBER Chapters* 3–46 (National Bureau of Economic Research, Inc, 1969).

10. Mincer, J. A. & Zarnowitz, V. The Evaluation of Economic Forecasts. in *Economic Forecasts and Expectations: Analysis of Forecasting Behavior and Performance* 3–46 (National Bureau of Economic Research, 1969).
